# Hybrid Deep Neural Network Scheduler for Job-Shop Problem Based on Convolution Two-Dimensional Transformation

**DOI:** 10.1155/2019/7172842

**Published:** 2019-07-10

**Authors:** Zelin Zang, Wanliang Wang, Yuhang Song, Linyan Lu, Weikun Li, Yule Wang, Yanwei Zhao

**Affiliations:** ^1^College of Computer Science and Technology, Zhejiang University of Technology, Hangzhou 310027, China; ^2^College of Science, Changchun University of Science and Technology, Changchun 130022, China; ^3^School of Engineering Science, King's College London, London WC2R 2LS, UK

## Abstract

In this paper, a hybrid deep neural network scheduler (HDNNS) is proposed to solve job-shop scheduling problems (JSSPs). In order to mine the state information of schedule processing, a job-shop scheduling problem is divided into several classification-based subproblems. And a deep learning framework is used for solving these subproblems. HDNNS applies the convolution two-dimensional transformation method (CTDT) to transform irregular scheduling information into regular features so that the convolution operation of deep learning can be introduced into dealing with JSSP. The simulation experiments designed for testing HDNNS are in the context of JSSPs with different scales of machines and jobs as well as different time distributions for processing procedures. The results show that the MAKESPAN index of HDNNS is 9% better than that of HNN and the index is also 4% better than that of ANN in ZLP dataset. With the same neural network structure, the training time of the HDNNS method is obviously shorter than that of the DEEPRM method. In addition, the scheduler has an excellent generalization performance, which can address large-scale scheduling problems with only small-scale training data.

## 1. Introduction

Job-shop scheduling problem (JSSP) [1] is one of the most famous problems in the industrial production, and it is categorized as a large class of intractable numerical problems known as NP-hard [2]. The solution space for an *m∗n* JSSP (where *m* is the number of machines and *n* is the number of jobs) is (*n*!)^*m*^ [3].

As it will be discussed in Section 2, many scholars have tried to solve this type of problems with population-based methods [4], gene-based methods [5], and heuristic methods [6]. However, in the face of large-scale problems, the response rate of the above methods has no distinct advantages. Many current researches show that data mining and machine learning methods have great potential in effect and efficiency [7]. In this paper, a hybrid deep neural network scheduler (HDNNS) is put forward to promote the scheduling capability. And convolution two-dimensional transformation (CTDT) is developed to convert JSSP's state information into regular information so that the process can be simplified in the convolutional network.

HDNNS has contributions in the following aspects:

- Based on the work of Weckman [3], Metan et al. [8], and Paolo et al. [9], HDNNS transforms JSSP into several classification subproblems. HDNNS's main innovation is the classification of the processing sequence of each job on each machine. The more precise classification method makes HDNNS more effective in the large-scale problems.
- Convolution two-dimensional transformation (CTDT) comes up in this paper. The function of CTDT is to convert the irregular scheduling data into regular multidimensional data with the form of Cartesian product. The transformed multidimensional data can be effectively processed in the deep convolution networks.
- HDNNS designs a hybrid neural network combining the deep convolution network [10] and the BP neural network [11]. In the first half of the network structure, convolution network and BP network are used to deal with the structural features and irregular features, respectively. After a certain number of layers of network processing, HDNNS merges these two networks with flattening operation for further feature extraction.

Our experimental results prove that the scheduling results of HDNNS are superior to many learning-based methods (ANN, HNN, and reinforcement learning methods), traditional classification methods (SVM, GOSS, and others), and attribute-oriented induction methods (AOI) [9] for the MAKESPAN index. HDNNS can occupy an advantage in the JSSPs compared with population-based methods (GA) and optimization methods (BBM). The value of HDNNS is not negated in the tests because GA and BBM are time consuming in computation. Besides, unlike GA and BBM, the HDNNS method has strong generalization performance. Our experiments certificate that a model trained by the data of small-scale JSSPs can address a large-scale one.

Although the training of the model requires extra time, the training process can be finished in advance. When the application environment remains stable, the model may not even need further updates. Such characteristics can increase the application value of the model to a certain extent. The training process can also be effectively accelerated by hardware such as GPU. Also, with the appropriate hardware (such as GPU and FPGA), the training speed will be significantly boosted.

The structure of this paper is as follows. In Section 2, a part of the most related work on the solution methods for JSSP has been reviewed along with neural network and other approaches available in the literature. In Section 3, the mathematical model of JSSP is proposed. In Section 4, the framework of the HDNNS is introduced, which includes scheduler structure, convolution two-dimensional transformation, and the basis of deep neural network. In Section 5, a 6 *∗* 8 JSSP example is applied to explain our method. In Section 6, six experiments are utilized to test the effectiveness and the generalization performance of the proposed method.

## 2. Related Works

### 2.1. Population-Based and Gene-Based Methods for JSSP

Over the last decades, JSSP has attracted much attention in the academia. Hence, a wide range of approaches have been developed for JSSP. Recently, population-based and gene-based methods are investigated to find optimal or near-optimal solutions.

Zhao et al. [12] proposed an improved particle swarm optimization with a decline disturbance index to improve the ability of particles in exploring global and local optimum solutions and to reduce the probability of particles being trapped into a local one. Peng et al. [13] combined a tabu search procedure with path relinking and showed that their method had a high performance in solving benchmark problem instances. Asadzadeh [14] tried to improve the efficiency of the genetic algorithm in solving JSSP by parallelizing populations and using an agent-based approach. Kurdi et al. [15] presented a modified island model genetic algorithm (IMGA) for JSSP. In this model, a nature-inspired evolutionary method and a migration selection mechanism have been added to the classical IMGA to improve diversification and delay premature convergence. Park et al. [16] proposed a dynamic JSSP and applied genetic programming-based hyper-heuristic methods with ensemble combination schemes to solve it. The investigated schemes had majority voting, linear combination, weighted majority voting, and weighted linear combination. It was concluded from the experiments that for the dynamic JSSP, the linear combination outperformed the other methods. Jiang et al. [17] employed the grey wolf optimization (GWO) to deal with two combinatorial optimization problems in the manufacturing field: job-shop and flexible job-shop scheduling cases. The discrete GWO algorithm was compared with other published algorithms for two scheduling cases. Experimental results demonstrate that our algorithm outperforms other algorithms for the scheduling problems under study. Fu et al. [18] proposed a fireworks algorithm with special strategies to solute the flow-shop scheduling problem under the consideration of multiple objectives, time-dependent processing time, and uncertainty. Sharma et al. [19] developed a variant of the ABC algorithm inspired from beer froth decay phenomenon to deal with job-shop scheduling problems.

There is no doubt that population-based and gene-based strategies are effective to solve JSSPs. However, faced with large-scale problems, the number of repeated iterations and updating operations often take a long time. Therefore, it is of great value to study a learning-based scheduler with fast response.

### 2.2. Learning-Based and Neural Network-Based Methods for Solving JSSP

With the further development of machine learning, some scholars try to solve JSSPs with learning-based methods. In this field, researches can be divided into two categories.

In the first category, learning methods are used to optimize population-based and gene-based methods. Learning methods optimize the updates of solutions, which thus improve the efficiency of optimization. Yang and Lu et al. [20] proposed a hybrid dynamic preemptive and competitive NN approach called the advanced preventive competitive NN method. A CNN was used to classify the system conditions into 50 groups. For each production interval, the current system status group was determined by CNN. Shiue et al. [21] extended the previous work by considering both the input control and the dispatching rule, such as those in a wafer fabrication manufacturing environment. In a novel recent work by Mirshekarian and Sormaz [22], a statistical study of the relationship between JSSP feature and optimal MAKESPAN was conducted. Ramanan et al. [23] proposed an artificial neural network-based heuristic method. This method utilized ANN to generate a solution of JSSP and then took it as the initial sequence to a heuristic proposed by Suliman. Adibi et al. [24] used a trained artificial neural network (ANN) to update parameters of a metaheuristic method at any rescheduling point in a dynamic JSSP according to the problem condition. Maroosi et al. [25] proposed an approach which utilizes the parallel membrane computing method and the harmony search method to solve flexible job shop problems. Information from the best solutions was used to boost the speed of convergence while preventing premature convergence to a local minimum.

In the second category, a reinforcement learning or machine learning framework is applied to build a learning-based model (ANN [11], SVM [26], CNN [27], or others). Then, the model is trained to master scheduling rules and complete automatic scheduling tasks. Weckman et al. [3] developed a neural network (NN) scheduler for JSSP in which the genetic algorithm was used to generate optimal or near-optimal solutions for a benchmark problem instance, and then, an NN was used to capture the predictive knowledge regarding the sequence of operations. Chen et al. [28] proposed a rule-driven dispatching method based on data envelopment analysis and reinforcement learning for the multiobjective scheduling problem. Mao et al. [29] presented the deep reinforcement learning method (DEEPRM) and translated the problem of packing tasks with multiple resource demands into a learning problem. This solution has an essential inspiration for solving the JSSP. Moreover, the initial results show that DEEPRM performs comparably to state-of-the-art heuristics, adapts to different conditions, converges quickly, and learns strategies that are sensible in hindsight. Shahrabi et al. [30] proposed a reinforcement learning (RL) with a Q-factor algorithm to enhance the performance of the scheduling method proposed for dynamic JSSP which considered random job arrivals and machine breakdowns. Nasiri et al. [31] used discrete event simulation and multilayer perceptron artificial neural network to solve the open-shop scheduling problem. Mohammad et al. [9] proposed a data mining-based approach to generate an improved initial population for population-based heuristics solving the JSSP. This method applied a combination of “attribute-oriented induction” and “association rule mining” techniques to extract the rules behind the optimal or near-optimal schedules of JSSP. Finally, their experiments verify the significant amount of FEs that can be saved using the proposed approach and the superiority of the proposed method in comparison with the method of Koonce and Tsai [32].

According to the retrospective literature, none of the previous studies directly applied deep learning frameworks to JSSP. This paper creates a convolution two-dimensional transformation and designs network structure to solve JSSP.

## 3. Mixed Integer Programming Model of JSSP

Job-shop scheduling problem (JSSP) can be described as a mixed integer programming problem.

The mathematical description is [33]

(1) $$\begin{array}{c} \text{min}:\;C_{\text{max}}, \end{array}$$

(2) $$\begin{array}{c} \text{s.t.}:\underset{j\in J}{\sum }x_{ijk}=1,\;\forall i\in M,k\in \left\{1,\cdots ,n\right\}, \end{array}$$

(3) $$\begin{array}{c} \overset{n}{\underset{k=1}{\sum }}x_{ijk}=1,\;\forall j\in J,i\in M, \end{array}$$

(4) $$\begin{array}{c} h_{ik}+\underset{j\in J}{\sum }p_{ij}x_{ijk}\leq h_{i,k+1},\;\forall i\in M,k\in \left\{1,\cdots ,n\right\}, \end{array}$$

(5) $$\begin{array}{c} \underset{i\in M}{\sum }r_{ijl}h_{ik}+\underset{i\in M}{\sum }r_{ijl}p_{il}\leq V\cdot \left(1-\underset{i\in M}{\sum }r_{ijl}x_{ijk}\right)+V\cdot \left(1-\underset{i\in M}{\sum }r_{ij,l+1}x_{ijk^{\prime }}\right)+\left(\underset{i\in M}{\sum }r_{ij,l+1}h_{ik^{\prime }}\right),\forall j\in J,i\in M,k,k^{\prime }\in \left\{1,\cdots ,n\right\},l\in 1,2,\cdots ,m-1, \end{array}$$

(6) $$\begin{array}{c} h_{in}+\underset{i\in J}{\sum }p_{ij}x_{ijk}\leq C_{\text{max}},\;\forall i\in M, \end{array}$$

(7) $$\begin{array}{c} h_{ik}\geq 0,\;\forall i\in M,k\in \left\{1,\cdots ,n\right\}, \end{array}$$

(8) $$\begin{array}{c} x_{ijk}\in \left\{0,1\right\},\;\forall i\in M,j\in J,k\in \left\{1,\cdots ,n\right\}. \end{array}$$

The decision variables are defined as follows:

- *x*_*ijk*_ is equal to 1 if job *j* is scheduled at the *k*-th position on machine *i*
- *h*_*ik*_ denotes the start time of the job at the *k*-th position of machine *i*

The parameters are defined as follows:

- *J* is the set of the jobs, and *M* is the set of the machines
- *n* is the number of the jobs, and *n*=*card*(*J*)
- *m* is the number of the machines, and *m*=*card*(*M*)
- *p*_*ij*_ is a non-negative integer which represents the processing time of job *j* and machine *i*
- *r*_*ijk*_=1 if the *k*-th position of job *j* requires machine *i*

The objective function is in (1). Constraint (2) ensures that each position on each machine is assigned to exactly one job. Constraint (3) ensures that each job only gets one position on a machine. Constraint (4) states that the start time of a job on a machine should be larger than the completion time of the job scheduled at the previous position. Constraint (5) is the precedence constraint. It ensures that all operations of a job are executed in the given order. In (5), *V* is ∑_*i*∈*J*_∑_*i*∈*M*_*p*_*ij*_ since the completion time of any operation cannot exceed the summation of the processing times from all the operations. Constraint (6) ensures that the MAKESPAN is at least the largest completion time of the last job on all machines. Constraint (7) ensures that the start time of all jobs at all positions is greater or equal to 0.

## 4. Hybrid Deep Neural Network Scheduler

### 4.1. Scheduler Structure

A hybrid deep neural network scheduler (HDNNS) is designed based on convolution two-dimensional transformation (CTDT) and hybrid deep neural network.

**Figure 1:**
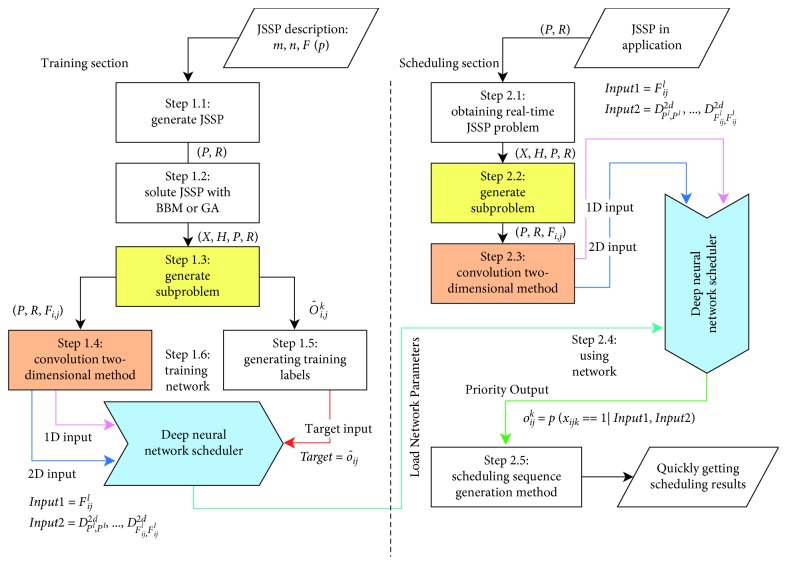
Structure of HDNNS. The contributions of this paper are reflected in the yellow, red, and blue structures.

The structure of the scheduler is shown in Figure 1.

HDNNS is divided into two sections: training section and scheduling section.

The training section has six steps (Step 1.1–Step 1.6). First, a large number of JSSPs are generated according to the JSSP description in Step 1.1. The description includes the number of machines *m*, the number of jobs *n*, and the distribution function of processing time *F*(*p*). Next, the generated problems are solved by state-of-the-art methods (BBM or GA in this paper). Moreover, corresponding scheduling results are generated in Step 1.2. In Step 1.3, each JSSP is divided into several subproblems, described as the features of a job processing and the priority in the machine. Features of job processing generate the 1D and 2D input data with CTDT in Step 1.4. Moreover, the priority in the machine generates onehot target data in Step 1.5. Finally, the scheduler training is in Step 1.6.

The training section has five steps (Step 2.1–Step 2.5). First, Step 2.1 is started when a new JSSP requires to schedule. Then, 1D input and 2D input can be produced by generating subproblem operation (same as Step 1.3) and convolution two-dimensional transformation operations (same as Step 1.4). In Step 2.4, we use a trained neural network to obtain the priority of each process in each job corresponding to the input of two groups of the neural network. In Step 2.5, a complete scheduling result is created with all priority results taken into account.

### 4.2. Mathematical Representation of Standard Solver and Division of Subproblems

Combined with the MIP description of JSSP in Section 3, all solvers are abstracted as follows:

(9) $$\begin{array}{c} X,H=S\left(P,R\right). \end{array}$$

In (9), *X* is the set of 0-1 decision variables *x*_*ijk*_, *H* is the set of integer decision variables *h*_*ik*_, *P* is the set of processing time data *p*_*ij*_, and *R* is the set of operation requiring data *r*_*ijk*_. And *S*(·) can be any scheduler for JSSP, such as genetic algorithm (GA) [14], branch and bound method (BBM) [34], and tabu search algorithm [13].

In order to improve the generalization performance of the model, HDNNS classifies a complete JSSP into several subproblems. Specifically, each subproblem determines the priority category on machine of the job processing process:

(10) $$\begin{array}{c} {\hat{A}}_{ij}=\underline{S}\left(P,R,F_{ij}^{*}\right). \end{array}$$

In (10), *F*_*ij*_^*∗*^ is the processing feature of job *j*'s *k*-th position in machine *i* and the relationship between the job's position and the machine is given by *R*. $\underline{S}\left(\cdot \right)$ is a subproblem scheduler from the *S*(·) in (9), and ${\hat{A}}_{ij}\in \left\{1,2,\cdots ,n\right\}$ is the integer priority of job processing on the machine (if in schedule result *X*, job *j* is processed in the order *k* in machine *i*, then *A*_*ij*_=*k*). The generation of *F*_*ij*_^*∗*^ and *A*_*ij*_ will be introduced in Sections 4.3 and 4.4.

**Figure 2:**
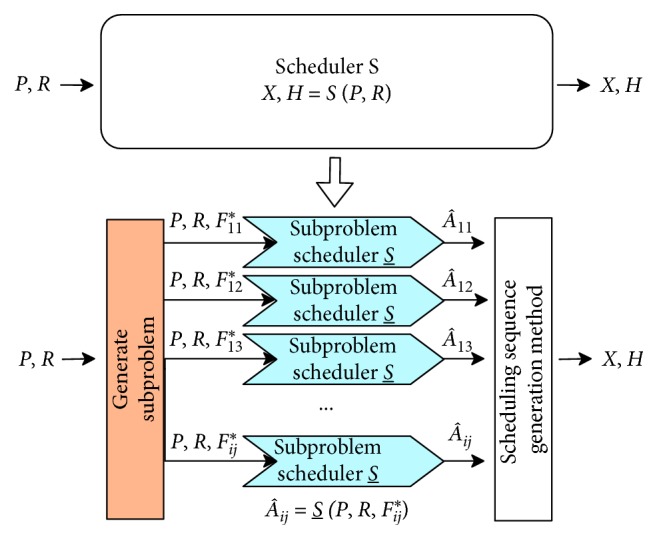
Generation of the subproblem.

The subproblem generation process is shown in Figure 2.

### 4.3. Convolution Two-Dimensional Transformation

#### 4.3.1. Definition of One-Dimensional Features

This paper designs a convolution two-dimensional transformation (CTDT) to extract scheduling features. Convolution operation is commonly used to extract features in the field of artificial intelligence and image processing [35, 36]. Many scholars believe that deep convolution operation is an effective way to extract complex combined features [37]. The CTDT is proposed to transform the irregular data in scheduling process (which cannot be convoluted directly) into regular data by the form of Cartesian product.

First, we define the 1-dimensional matrix relative machine processing time *p*^*l*^ from *P* as

(11) $$\begin{array}{c} P^{l}=\left[T_{1,1},T_{1,2},\cdots ,T_{1,j_{2}},\cdots ,T_{1,n},\cdots ,T_{j_{1},j_{2}},\cdots ,T_{n,n}\right]. \end{array}$$

In (11), *T*_*j*_1_,*j*_2__, *j*_1_, *j*_2_ ∈ *J* is the ratio of processing time of job *j*_1_ to that of job *j*_2_, which are represented as follows:

(12) $$\begin{array}{c} T_{j_{1},j_{2}}=\frac{\sum_{i\in J}p_{ij_{1}}}{\sum_{i\in J}p_{ij_{2}}}, \end{array}$$

*P*^*l*^ will provide the scheduler with relative information about the processing time of jobs.

Then, we define the 1-dimensional matrix's earliest start time *E*^*l*^ from *P* and *R* as

(13) $$\begin{array}{c} E^{l}=\left[e_{1k},e_{2k},\cdots ,e_{jk},\cdots ,e_{nk}\right]. \end{array}$$

In (13), *e*_*jk*_, *j* ∈ *J*, *k* ∈ {1,2, ⋯, *n*}, is the earliest start time of job *j*'s *k*-th position shown as follows:

(14) $$\begin{array}{c} e_{jk}=\underset{i\in M,k^{*}\in \left\{1,2,\cdots ,k-1\right\}}{\sum }r_{ijk^{*}}p_{ijk^{*}}. \end{array}$$

In (14), *P*^*l*^ provides the urgency information of jobs.

Similarly, we define the 1-dimensional other features *F*_*ij*_^*l*^ as

(15) $$\begin{array}{c} F_{ij}^{l}=\left[f_{ij,1}^{*},f_{ij,2}^{*},\cdots ,f_{ij,N_{f}}^{*}\right]. \end{array}$$

In (15), *F*_*ij*_^*l*^ consists of a series of important features in reference and application. *N*_*f*_ is the number of the features, and in this paper, *N*_*f*_=10. The features are given in Table 1.

In Table 1, the variables in tables are defined as follows:

(16) $$\begin{array}{c} T^{\text{total}}=\underset{i\in M,j\in J}{\sum }p_{ij}, \end{array}$$

(17) $$\begin{array}{c} T_{i}^{\text{cmp}}=\underset{i\in M}{\sum }p_{ij}, \end{array}$$

(18) $$\begin{array}{c} T_{j}^{\text{cjp}}=\underset{j\in J}{\sum }p_{ij}. \end{array}$$

In (16)–(18), *T*^total^ is the total processing time, *T*_*i*_^cmp^ is the processing time of machine *i*, and *T*_*j*_^cjp^ is the processing time of job *j*.

#### 4.3.2. Convolution Two-Dimensional Transformation and Definition of Two-Dimensional Matrix

Cartesian product operation can combine linear features and convert one-dimensional feature data into two-dimensional feature data. This paper designs convolution two-dimensional transformation (CTDT) based on Cartesian product.

The transformation is described in

(19) $$\begin{array}{c} D_{m_{1}^{l},m_{2}^{l}}^{2d}=T\left(m_{1}^{l},m_{2}^{l},\alpha ,\beta \right)=\text{sigmoid}\left(\alpha \cdot {\left(m_{1}^{l}\times m_{2}^{l}\right)}^{\beta }\right), \end{array}$$

(20) $$\begin{array}{c} m_{1}^{l}\times m_{2}^{l}=\left\{x\cdot y\left|x\right.\in m_{1}^{l},\wedge y\in m_{2}^{l}\right\}. \end{array}$$

In (19), *m*_1_^*l*^ and *m*_2_^*l*^ are the two one-dimensional features and × is the sign of Cartesian product; the mathematical definition is shown in (20). *α* and *β* are the parameters of this transformation. sigmoid(·) is a nonlinear activation function. This function will match the model parameters and extract new features in different horizons. The sigmoid function is shown in

(21) $$\begin{array}{c} \text{sigmoid}\left(x\right)=\frac{1}{1+e^{-x}}. \end{array}$$

In (21), *x* is a matrix.

**Figure 3:**
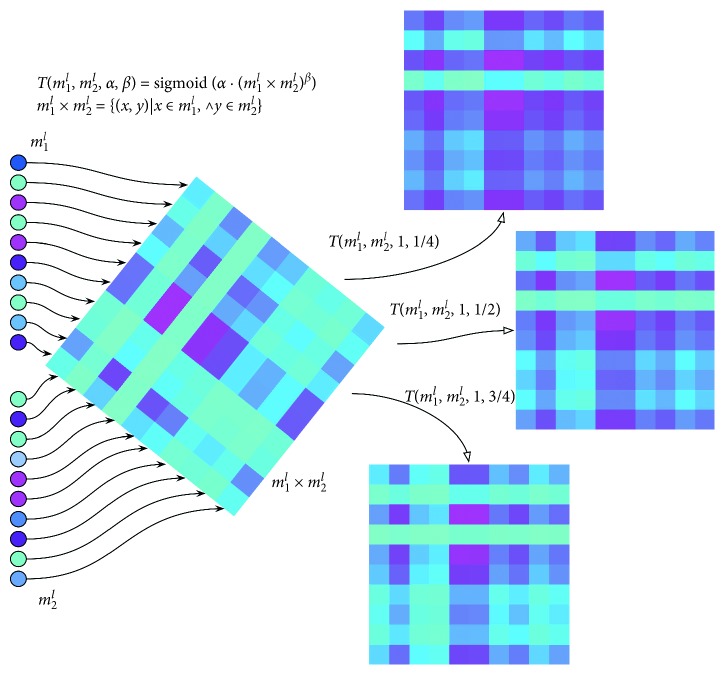
Concise sketch map of linear structure.

An example of a *T*(·) function is shown in Figure 3.

In Figure 3, *m*_1_^*l*^ and *m*_2_^*l*^ are the two one-dimensional data like *P*^*l*^, *E*^*l*^, and *F*_*ij*_^*l*^ in Section 4.3.1. The Cartesian product of *m*_1_^*l*^ and *m*_2_^*l*^ is *m*_1_^*l*^ × *m*_2_^*l*^. Three sets of parameters are used to normalize *m*_1_^*l*^ × *m*_2_^*l*^ in Figure 3. Different parameters mean that the model pays attention to different data scales, which helps the model to discover the characteristics of different scales.

### 4.4. Training Labels

HDNNS transforms the scheduling problem into classification problems. So, this paper uses onehot encoding [38] to define training labels.

**Figure 4:**
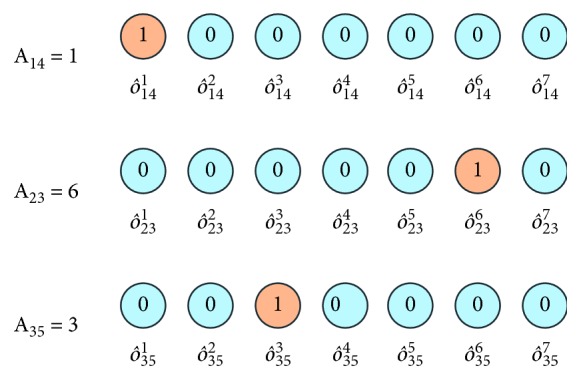
Concise sketch map of linear structure.

Job *i*'s *k*-th position (one machine *j*) onehot priority label ${\hat{o}}_{ij}^{k}$ is shown in (22). Three examples are given in Figure 4:

(22) $$\begin{array}{c} {\hat{o}}_{ij}^{k}=\left\{\begin{array}{c} 1,\;\text{if}\;\;k={\hat{A}}_{ij},\\ 0,\;\text{if}\;\;k\neq {\hat{A}}_{ij}. \end{array}\right. \end{array}$$

In (22), *A*_*ij*_ is the number of positions in job *i* machine *j*, defined in Section 4.2.

### 4.5. Structure of Hybrid Deep Neural Network Scheduler

In this section, an innovative hybrid deep neural network structure for JSSP is introduced.

As shown in Figure 1, the inputs of the hybrid deep neural network scheduler are one-dimensional input *Input*1, two-dimensional input *Input*2, and target input *Target*.

The expression is shown in

(23) $$\begin{array}{c} Input1=F_{ij}^{l},\\ Input2=\left[D_{P^{l},P^{l}}^{2d},D_{P^{l},E^{l}}^{2d},D_{P^{l},F_{ij}^{l}}^{2d},D_{E^{l},E^{l}}^{2d},D_{E^{l},F_{ij}^{l}}^{2d},D_{F_{ij}^{l},F_{ij}^{l}}^{2d}\right],\\ Target={\hat{o}}_{ij}. \end{array}$$

**Figure 5:**
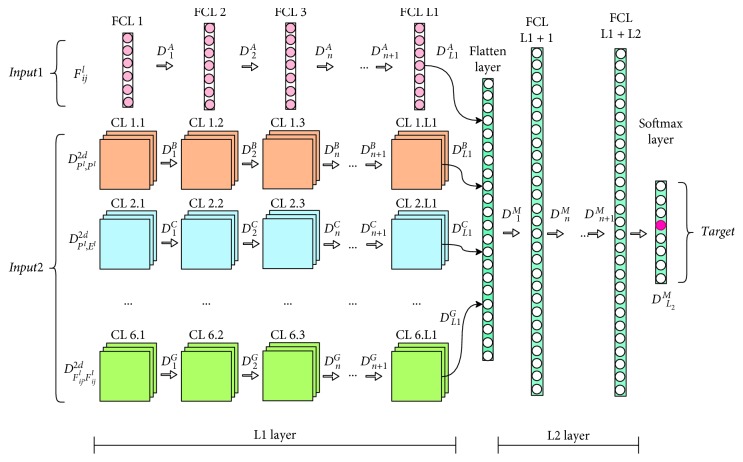
Schematic diagram of a hybrid deep neural network structure.

The general structure of the network is shown in Figure 5.

In Figure 5, the left side of the structure diagram is the input part of the network.

For *Input*1, HDNNS uses *L*1 layers (fully connected layer) [39] (FCL in the figure) to preliminarily extract one-dimensional features. As shown in Figure 5, the output of the *g*-th layer is defined as *D*_*g*_^*A*^ and the output of the final layer is *D*_*L*1_^*A*^. The fully connected layer is a typical combination of neurons in the deep convolution network [39].

For *Input*2, HDNNS uses *L*1 layers (convolutional layer) [39] (CL in the figure) to preliminarily extract two-dimensional features. The size of the convolution kernel [39] is set to 3 *∗* 3. As shown in Figure 5, the output of the *g*-th layer is defined as *D*_*g*_^*B*^ to *D*_*g*_^*G*^ in different features in (23) and the weight of the *g*-th layer is defined as *W*_*g*_^*B*^ to *W*_*g*_^*G*^.

At the *L*1+1th layer of the network, one-dimensional features and two-dimensional features are combined by flattening operation in the flattened layer [40], described as

(24) $$\begin{array}{c} D_{i,q}^{M}=\sum^{}W_{L1,q}^{A}\cdot D_{L1}^{A}+\sum^{}W_{L1,q}^{B}\cdot D_{L1}^{B}+\cdots +\sum^{}W_{L1,q}^{G}\cdot D_{L1}^{G}. \end{array}$$

In (24), *W*_*L*1,*q*_^*A*^, *W*_*L*1,*q*_^*B*^, ⋯, and *W*_*L*1,*q*_^*G*^ are the network weights of layers *FCLL*1, *CL*1.*L*1, ⋯, *CL*6.*L*1 and *q* is a neural index.

After *L*2 fully connected layers, the feature passes through a Softmax layer [39] containing only *n* neurons. This layer converts the feature signal into a meaningful probability description *o*_*ij*_. *o*_*ij*_ has the same shape with the target ${\hat{o}}_{ij}$. However, *o*_*ij*_ is not a 0-1 variable, but a continuous quantity, which satisfies *o*_*ij*_^*p*^ ∈ [0 − 1], where *p* ∈ {1,2, ⋯, *n*}. *o*_*ij*_^*p*^ can be interpreted as the possibility of selecting priority *p*.

After defining the structure of the neural network, we use the error backpropagation (BP) method [39] to train the network parameter.

A trained neural network can be described as a function mapping in the scheduling section of Figure 1, which is shown in the following formula:

(25) $$\begin{array}{c} o_{ij}=DNNS\left(Input1,Input2\right). \end{array}$$

In (25), *DNNS*(·) is the deep neural network scheduler and the input function is *Input*1 and *Input*2 in Figure 1 and (23). The *o*_*ij*_ is the possibility that the current subproblem belongs to each priority. There are *n* priorities, so there are *n* elements in *o*_*ij*_, each of which is described as

(26) $$\begin{array}{c} o_{ij}^{k}=p\left(x_{ijk}==1\left|Input1\right.,Input2\right),\\ k\in \left\{1,2,\cdots ,n\right\},\\ Input1=F_{ij}^{l},\\ Input2=\left[D_{P^{l},P^{l}}^{2d},D_{P^{l},E^{l}}^{2d},D_{P^{l},F_{ij}^{l}}^{2d},D_{E^{l},E^{l}}^{2d},D_{E^{l},F_{ij}^{l}}^{2d},D_{F_{ij}^{l},F_{ij}^{l}}^{2d}\right]. \end{array}$$

### 4.6. Scheduling Sequence Generation Method

In (10), the whole problem is decomposed into several subproblems. In this part, a scheduling sequence generation algorithm combines the solutions of the subproblems into a complete solution of JSSP.

The pseudocode description of the method is shown in Algorithm 1.

In Algorithm 1, each cycle for *i* will determine the scheduling order of one machine. Each cycle for *j* will determine the scheduling sequence of one job in the machine *i*.

When determining the order of jobs, in Step 7, the algorithm first chooses the most assured judgment of the neural network scheduler, and the most reliable judgment is the output probability closest to 1. In Step 8 and Step 9, when the job *I*_*j*_ is selected as priority *I*_*p*_, the other data of job *I*_*j*_ and priority *I*_*p*_ are set to 0 according to constraints (2) and (3) to avoid the conflict in the next loop. In Step 9, the algorithm updates the value of the output matrix.

### 4.7. Generalization Performance of HDNNS

HDNNS algorithm has a reliable generalization. Specifically, we can easily extend the training results of smaller-scale JSSPs (the number of machines is small) to solve larger-scale JSSPs (the number of machines is significant). Such characteristics give HDNNS a unique advantage. When the solution of a large-scale problem is difficult to be generated by the existing methods, HDNNS can use the solution of a small-scale problem to train the network and then use the trained model to schedule a large-scale problem.

In (26), the input parameters of the trained scheduler are composed of two sets of data, one of which is one-dimensional data and the other is two-dimensional data generated by CTDT. For all inputs, as the number of machines increases, the input and output structures of the neural network will not change.

Although the absolute value of the parameter changes, the correlation between the parameters still exists. The scheduler will use these features with relationship to complete the scheduling. Of course, the more significant the gap between the scale of training data and the scale of actual scheduling data, the bigger the error of results. This paper will discuss it in the experiment.

## 5. An Example of HDNNS

In this section, we illustrate HDNNS with an example (*m*=6, *n*=8).

In the training section of Figure 1, we generate a series of JSSP and solve them as the training data.

An example of algorithm generation of JSSP is described as Tables 2 and 3.

**Figure 6:**
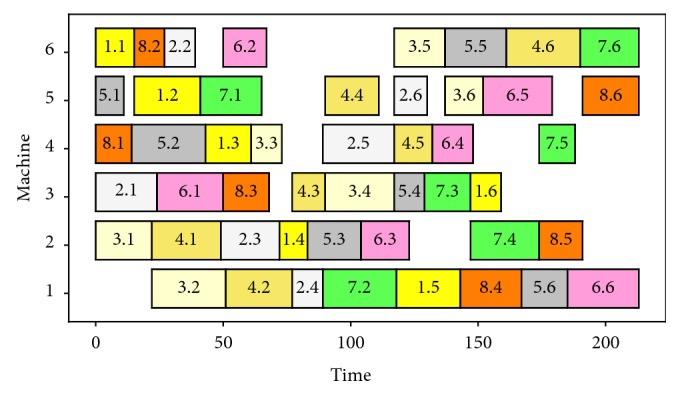
Gantt chart of current JSSP.

Tables 2 and 3 describe a 6 *∗* 8 JSSP, and Figure 6 shows the Gantt chart of the optimal solution (with BBM). In Table 2, the number in line *j* and column *k* is the time required for job *j*'s *k*-th position. In Table 3, the number in line *j* and column *k* is the machine required for job *j*'s *k*-th position.

In Figure 6, the horizontal axis is the time axis and the ordinate axis is the machines axis. Each block represents a processing, and different colors represent different jobs. The text *j* · *k* in the boxes means that the processing of job *j*'s *k*-th position starts at the time of the left side of the block and ends at the right side of the block.

Then, 48 subproblems are generated according to (10). One-dimensional and two-dimensional features are extracted for each subproblem, and training data such as (23) are generated in Table 4.

**Figure 7:**
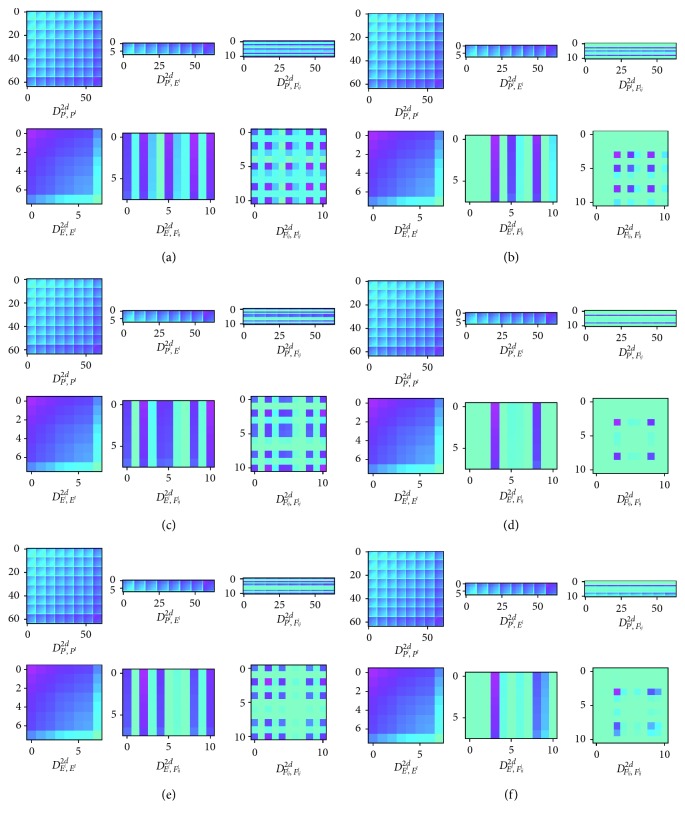
Feasibility analysis of CTDT. (a) Subproblem of *i*=3, *j*=1, *k*=6. (b) Subproblem of *i*=6, *j*=1, *k*=1. (c) Subproblem of *i*=5, *j*=2, *k*=6. (d) Subproblem of *i*=2, *j*=4, *k*=1. (e) Subproblem of *i*=6, *j*=7, *k*=6. (f) Subproblem of *i*=4, *j*=5, *k*=1.

Six groups of two-dimensional features are selected for visual display, and the pictures are shown in Figure 7.

Six groups of matrices generated by CTDT are shown in Figure 7. Among them, (a), (c), and (e) have a high priority and the other three have a low priority.

In this extreme case of the highest priority and the lowest priority, it is easy to find that images with the same priority have a lot in common. In general, the hue of matrix *D*_*P*^*l*^,*F*_*ij*_^*l*^_^2*d*^, *D*_*E*^*l*^,*F*_*ij*_^*l*^_^2*d*^, and *D*_*F*_*ij*_^*l*^,*F*_*ij*_^*l*^_^2*d*^ in (a), (c), and (d) is darker and that of matrix *D*_*P*^*l*^,*F*_*ij*_^*l*^_^2*d*^, *D*_*E*^*l*^,*F*_*ij*_^*l*^_^2*d*^, *D*_*F*_*ij*_^*l*^,*F*_*ij*_^*l*^_^2*d*^ in (b), (d), and (e) is brighter. The remaining three matrices describe the whole problem rather than the subproblem. Therefore, the same graphics are shown in different subproblems.

Although identifying similar priority categories is more difficult for human beings, our deep learning-based scheduler can effectively extract the priority information.

After training the network with the data in Table 4, we get a scheduler that can respond quickly. When a new scheduling problem arrives, the scheduler processes the problem according to (25) and gets the priority matrix *O*. For this problem, the output example of matrix *O* is shown in Table 5.

Finally, the scheduling sequence generation algorithm is used to process the output matrix and the scheduling order *X* and the time result in Figure 6 can be obtained.

## 6. Results and Discussion

### 6.1. Parameters and Effect Experiment

In this part, the training process of HDNNS and the influence of different parameters on HDNNS are discussed.

Dataset ZLP (7 *∗* 7) [41] is used in this part to validate the effectiveness of the method effectively. ZLP (7 *∗* 7) dataset contains 2000 7 *∗* 7 (*m*=7, *n*=7) JSSPs, and it corresponds to solutions.

**Figure 8:**
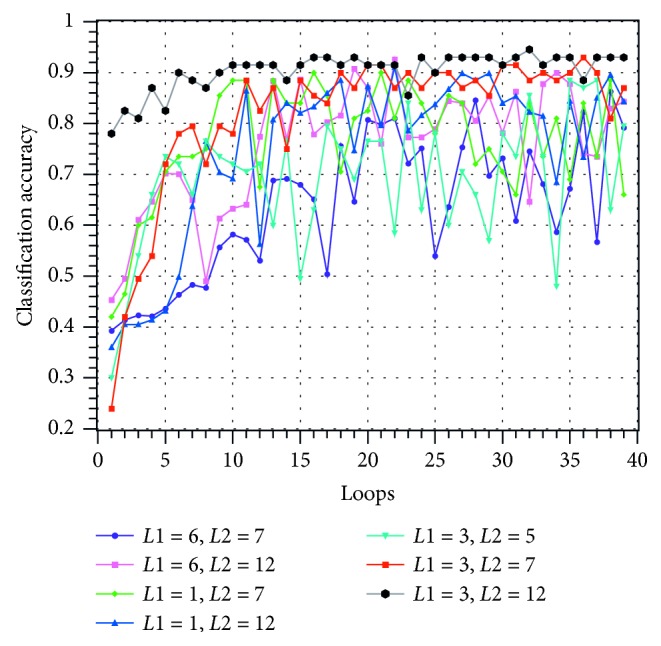
Classification accuracy change diagram during training under different parameters.

**Figure 9:**
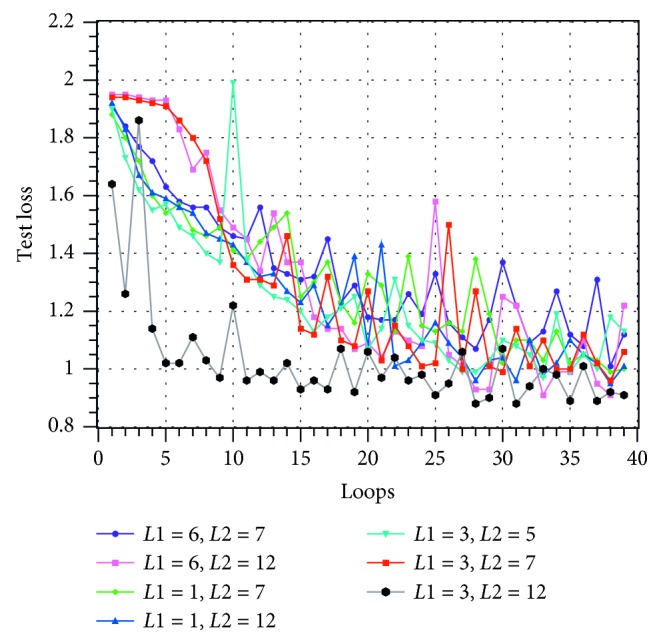
Loss change diagram during training under different parameters.

**Figure 10:**
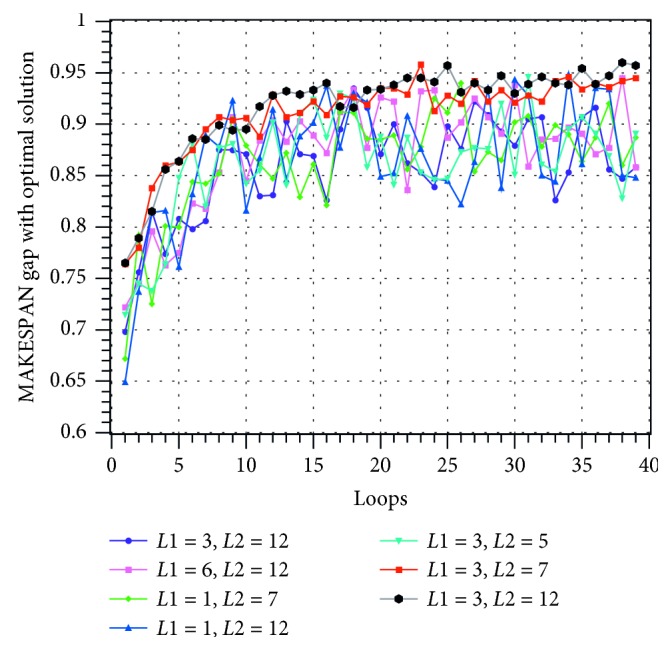
MAKESPAN change diagram during training under different parameters.

This experiment trains the scheduler with the first 1500 questions and labels and then tests the scheduler with the last 500 questions. The learning rate of the network is 0.01. The training process curve is plotted in Figures 8–10.

In Figures 8–10, the horizontal axis is the number of training loops and the vertical axis is the classification correctness, classification loss, and MAKESPAN [22] (completion time of processing). The curves of different colors represent the experimental results obtained by choosing different model parameters *L*1 and *L*2. Among them, the loss evaluation index calculation formula is

(27) $$\begin{array}{c} L\left(\hat{o},o\right)=-\log\;P\left(o\left|Input1\right.;Input2\right)\\ =\frac{1}{N_{\text{test}}}\overset{N_{\text{test}}}{\underset{c=1}{\sum }}\overset{n}{\underset{k=1}{\sum }}y_{ck}\log\left(o_{ck}\right). \end{array}$$

In (27), $\hat{o}$ is the target output, *o* is the probabilistic description of current features belonging to various classifications, and *L*(·) is the loss function. *y*_*ck*_ is the bool value, and this value indicates whether the target class of input features *Input*1, *Input*2 instance is *k*. *o*_*ck*_ is the probability of input features *Input*1, *Input*2 belonging to class *k* predicted by the model. There is a one-to-one mathematical relationship between *o*_*ck*_and *o*_*ij*_^*k*^ in (26).

The three figures show that the performance of the model improves gradually with the increase of the number of training cycles. This improvement can be achieved until the classification accuracy reaches more than 90% and the model loss reaches less than 1. Moreover, the disparity between the scheduling result and the optimal solution reaches less than 5%. The above experiments show that the HDNNS can effectively train the scheduler to complete the JSSP scheduling task.

Seven groups of different model parameters were selected and tested. The experimental results show that among all the parameters, *L*1=3 and *L*2=12 have better results. When *L*1=3 and *L*2=12, the classification accuracy of the centralized test is more than 93%, the loss is less than 85%, and the gap of MAKESPAN is less than 4%.

### 6.2. Confusion Matrix of the Result

In order to measure the effectiveness of HDNNS, this paper compares it with the classical ANN [3] method.

Dataset ZLP (7 *∗* 7) [41] is used in this part to train two kinds of neural networks. This experiment trains the scheduler with the first 1500 questions and labels. Then, this experiment tests the scheduler with the last 500 questions. For HDNNS, the size of the convolution kernel is 3 *∗* 3, and *L*1=3, *L*2=12, and learning rate is 0.01. For the ANN method, the ANN structure is 11-12-10-7 and learning rate is 0.01.

The classification confusion matrix of ANN and HDNNS (the output of Step 2.4 in Figure 1) is shown in Tables 6 and 7.

In Tables 6 and 7, the line *i* and column *j* is the number of times that the job with the *i*th position of the machine has been assigned to the *j*th position of the machine. The priority of job 2 in machine 1 is 2, meaning that this job is in the second position of machine 1's processing. If a scheduler classifies the location of job 2 in the machine 1 as 2, one will be added to the second row and the second column of the confusion matrix. If a scheduler classifies the location of job 2 in the machine 1 as 3, one will be added to the second row and the third column of the confusion matrix. Therefore, the larger the number on the diagonal line, the higher the accuracy of the model.

**Figure 11:**
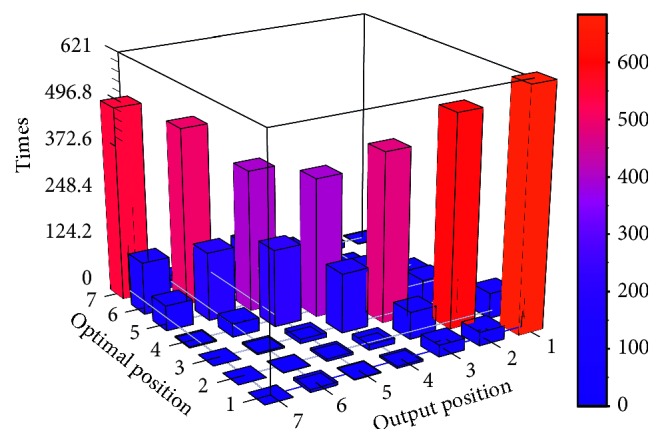
Confusion matrix bar graph of ANN.

**Figure 12:**
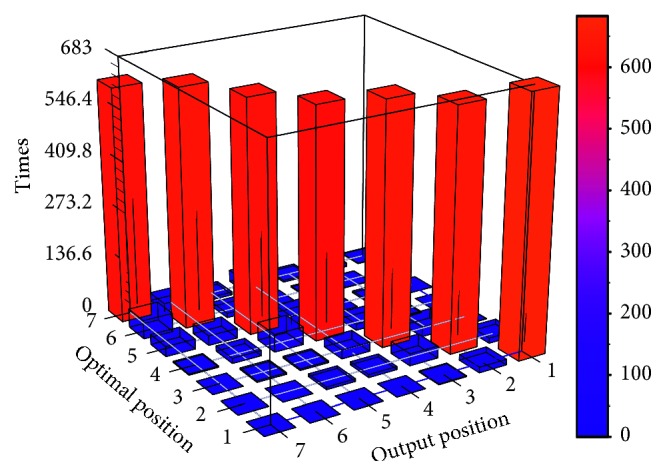
Confusion matrix bar graph of HDNNS.

The bar figure of the confusion matrix is shown in Figures 11 and 12.

Tables 6 and 7 and Figures 11 and 12 show that the classification performance of HDNNS is better than ANN. On the stability of classification, two methods can classify the highest and lowest priority jobs more accurately because the boundary of classification will introduce less noise interference. However, the classification accuracy of each priority of the HDNNS method is more stable. The accuracy of classification results of the HDNNS method fluctuates between 88% and 98%. In terms of classification accuracy, HDNNS can achieve 90% classification accuracy. It is better than 60% of the ANN method.

The essence of the learning-based method is to estimate the probability from input to output by finding the implicit relationship between them. Because the ANN method does not consider the depth of local features combination, its effect is not ideal. For example, the *p*_*ij*_ has no noticeable effect on its priority. However, the combination of *p*_*ij*_, the *p*_*ij*_/*T*_*i*_^cmp^ , and the *T*_*i*_^cmp^/*T*^total^ has a more significant impact on the final output.

The traditional neural network does not have strong ability to deal with combined features. Thus, ANN is difficult to achieve effective training because of the disappearance of the gradient [36]. In this paper, deep convolutional network is introduced into the scheduling problem to solve the problem of learning and training combined features, which improves the accuracy of network classification.

### 6.3. MAKESPAN and Time Consumption Comparisons in ZLP Dataset

JSSP scheduling methods are divided into two categories: population-based (gene-based) method and learning-based method. The population-based (gene-based) method obtains the near-optimal solution by updating the solutions set. The effect of this method is often better than the other two algorithms. Because iteration will produce a lot of time cost, this kind of method can often get excellent scheduling results. Therefore, this subsection does not compare population-based (gene-based) methods.

This subsection will discuss the performance of HDNNS algorithm from the above two aspects. This part tests the performance of deep reinforcement learning (DEEPRL) [29, 42], deep Q learning (DQN) [43], artificial neural network (ANN) [3], Hopfield neural network (HNN) [44], stochastic processing time (SHPT) [45] method, and shortest processing time (SPT) [46] method.

The dataset is ZLP dataset [41], which contains 2000 8 *∗* 8 JSSPs (*m*=8, *n*=8) and 13 *∗* 13 JSSPs (*m*=13, *n*=13). The solution of JSSPs above is generated with the BBM method [47]. The processing time matrix *p* of each JSSP satisfies the uniform distribution *U*(*a*, *b*). For HDNNS, ANN and the first 1500 JSSPs are used in the training section. The last 500 JSSPs are used in scheduling section to test the performance of the model. The DEEPRM method is trained by interacting with the JSSP model. The size of the convolution kernel is 3 *∗* 3, and *L*1=3 and *L*2=12. The learning rate of HDNNS, ANN, and DEEPRL is 0.01. The number of learning epochs is 100. For HNN, SHPT, and SPT, the performance of the method is tested directly with the last 500 data. The experimental environment is Lenovo k4450, Ubuntu 16, CPU i4700 2.1 MHZ, Python, and Tensorflow.

The results of the experiment are shown in Table 8. Four indexes are discussed in the table: average MAKESPAN, scheduling score, scheduling time, and training time. The scheduling score is calculated according to

(28) $$\begin{array}{c} S\left(A,D\right)=\frac{M\left(O_{\text{ptimal}},D\right)}{M\left(C_{\text{urrent}},D\right)}. \end{array}$$

In (28), the scheduling capacity *S*(*A*, *D*) of current algorithm *A* in database *D* is defined as the ratio of the average optimal MAKESPAN *M*(*O*_ptimal_, *D*) to the current algorithm's MAKESPAN *M*(*C*_urrent_, *D*).

Eight groups of JSSPs are tested in Table 8. The first four groups are 8 *∗* 8 JSSPs, and the last four groups are 13 *∗* 13 JSSPs. Each problem is generated by random-based function, and its processing time satisfies the uniform distribution *p* ~ *U*(*a*, *b*).

For the learning-based method (HDNNS, DQN, DEEPRL, and HNN), the time consumption is divided into training time and scheduling time. The training time is the total time needed for 100 epochs of model training. The scheduling time recorded the total time of testing 500 JSSPs.

The ANN method is tested in two cases, one (ANN (1D) in proposed in [3]) using only one-dimensional feature as the input feature and the other (ANN (ALL)) using flattened one-dimensional features and two-dimensional features as input features. ANN (1D) has a smaller network structure, so it has faster training efficiency and scheduling efficiency. Although the scheduling results of ANN (ALL) are better than that of ANN (1D), its training time is significantly improved with the JSSP scale. It is because the network structure of ANN has no advantage in dealing with complex scheduling information. Moreover, it cannot adequately deal with the relationship between the combination features and the output. In general, the scheduling effect of ANN network is better than the SPT method and STPT method.

Hopfield neural network (HNN) can also effectively obtain the scheduling results. But, unlike ANN, HNN seeks stability point through evolution and achieves the purpose of scheduling. HNN has a good effect on small-scale problems but suffers from the resolution of large-scale problems.

DEEPRM and DQN are scheduling methods based on reinforcement learning (DEEPRM's network structure is the same as that of HDNNS, and DQN use a standard deep network). These methods do not need labeled training data in the training section, but they need much interaction with the scheduling environment. In most cases, interaction learning is much slower than learning through training data. For JSSP which has easy access to label data, DEEPRM and DQN have disadvantages in training efficiency.

HDNNS is stable in different processing time distributions *p* ~ *N*(*a*, *b*) and different problem scales *m* and *n*. Moreover, the scheduling ability is maintained at 90% of the optimal solution, which is superior to the same ANN and HNN. Although the training time of HDNNS is longer than that of ANN (1D), it does not affect the real-time scheduling of the scheduler in applications because the training phase can be completed beforehand. Considering the scheduling performance of all the algorithms, HDNNS has significant advantages.

### 6.4. MAKESPAN and Time Consumption Comparisons in Traditional Dataset

This subsection uses the same methods as in Section 6.3 to solve the classical JSSPs, which include ft10 [48], ft20 [48], la24 [49], la36 [49], abz7 [50], and yn1 [51].

The experimental procedure is as follows. First, 2000 JSSPs of the same scale as the under test JSSP are generated. Then, the state-of-the-art method is used to find the optimal solution (near-optimal solution) as the training data. In this experiment, the solution of smaller JSSP (ft10, ft20, la24) is generated by the BBM method [47]. Moreover, the solution of larger JSSP (la36, abz7, yn1) is generated by the GA method. The first 1500 JSSPs are used in the training section. The last 500 JSSPs are used in scheduling section to test the performance of the mode.

The DEEPRM method is trained by interacting with the JSSP model. The learning rate of HDNNS, ANN, and DEEPRM is 0.01. The number of learning epochs is 100. The experimental environment is Lenovo k4450, Ubuntu 16, and CPU i4700 2.1 MHZ.

The test results are shown in Table 9.

The structure of Table 9 is the same as that of Table 8. The first column shows the optimal solution. Six popular JSSPs are tested in Table 9. The brackets below the JSSP name indicate the size of the problem.

Testing with separate test questions introduces randomness, so we recommend using the average of a large number of test results to measure the effectiveness of the algorithm (like ZLP datasets).

### 6.5. MAKESPAN Comparisons with Traditional Classification Algorithms

In this subsection, several traditional classification methods are used to compare with HDNNS. HDNNS is essentially a classification-based method, so it is necessary to compare it with some traditional classification methods. We replace the deep neural network scheduler in Figure 1 with other classification methods and measure its effect.

In this experiment, we test *k*-nearest neighbor (KNN) [52], support vector machine (SVM) [26], decision tree (DT) [53], extremely randomized trees (ERT) [54], and Gaussian model (GOSS) [55].

The test dataset is ZLP dataset [41], which contains 2000 15 *∗* 12 JSSPs (*m*=15, *n*=12) and 2000 15 *∗* 18 JSSPs (*m*=15, *n*=18). The solution of JSSPs above is generated with the GA method. The first 1500 JSSPs are used as the training section. Then, the performance of the method is tested with the last 500 data. The parameters of the above classification methods are the default parameters of Python 3's sklearn tool kit. The result of the solution is shown in Table 10.

The experimental results show that HDNNS has a significant advantage over traditional classification algorithms. Although the traditional method has an advantage in efficiency, it can only achieve the 80% of near-optimal solution. Therefore, HDNNS has a big advantage in the framework of this paper.

### 6.6. Analysis of Generalization Performance

HDNNS has a good scalability, and a trained scheduler can be used to solve problems of different machine numbers *m*. In other words, models trained with less complex problems can be used to solve more complex problems. Based on this premise, it is necessary to measure the generalization of models at different levels of complexity.

This subsection discusses the performance of models trained with small-scale data in solving large-scale problems. In the experiment, 1500 JSSPs (labels are generated with GA) are used as the training section. Then, groups of larger problems (larger machine number *m*) are applied to test the scheduling capability of HDNNS. In order to get a credible conclusion, the experiment generates 500 different JSSPs and corresponding near-optimal solution for each group.

**Figure 13:**
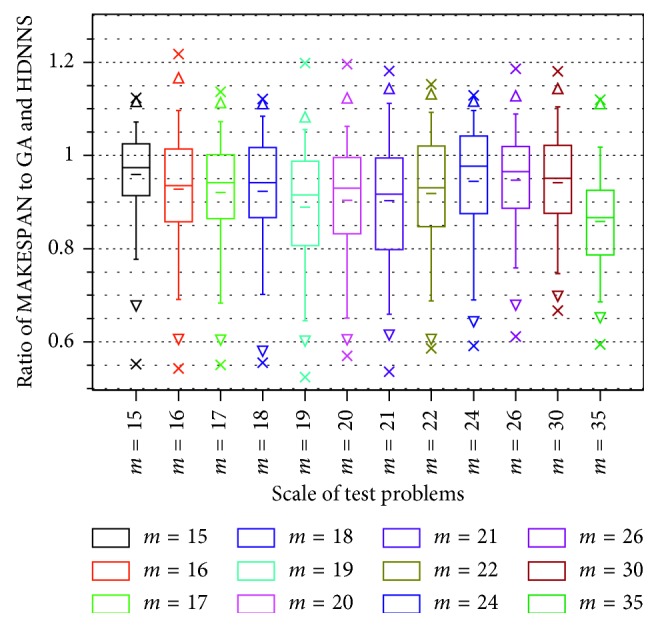
Box diagram of testing the model trained by 15 *∗* 15 dataset with a larger scale problem.

The box diagram of the experiment is shown in Figure 13.

In Figure 13, each box in the diagram represents a test result of a group. The top and bottom multiplication symbols represent the maximum and minimum values in the test. Moreover, the top and bottom triangles between the multiplication symbol is the 1% point and the 99% point of the 200 data. The lower and upper bounds of the boxes are 25% and 75% of the 200 data. The horizontal longer line in the middle of the box is the median number, and the horizontal shorter line is the average number.

Figure 13 shows that the closer the scale of test problems and training problems is, the better their performance wil l be. The average ratio of MAKESPAN obtained by HDNNS to GA is 0.97, and the MAKESPAN of the scheduling result is also stable.

With the increase in the number of machines, the model's efficiency gradually decreases, which is embodied in the decline of the excellent degree of the solution and the stability of the solution. However, the decline in solving ability is not rapid and unacceptable.

We are happy to see that our scheduler can extract scheduling knowledge from a simple JSSP and use it successfully in a more complex scheduling problem. Specifically, the excellent degree of solutions of all test problems is greater than 0.86 (average).

## 7. Conclusion

A hybrid deep neural network scheduler with the characteristics of offline training and online real-time scheduling is created in this paper. In this scheduler, we present two innovations based on the machine learning framework. One is the convolution two-dimensional transformation (CTDT), which converts the irregular data in the scheduling process into regular data; this enables deep convolutional operation to be used to solve JSSP. Another is hybrid deep neural network structure including convolution layer, fully connected layer, and flattening layer. And, this structure can effectively complete the extraction of scheduling knowledge.

The results show that the MAKESPAN index of HDNNS is 9% better than that of HNN and is 4% better than that of ANN in ZLP dataset. The training time of the HDNNS method is obviously faster than that of the DEEPRM method with the same neural network structure. Besides, the scheduler has brilliant generalization ability, which can solve large-scale scheduling issues with small-scale training data.

## Figures and Tables

**Algorithm 1:**
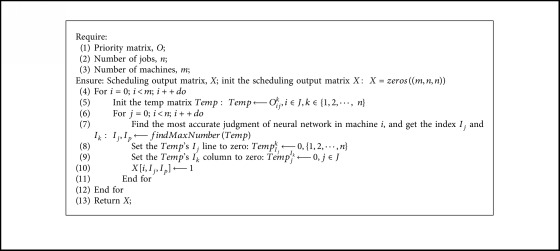
Scheduling sequence generation algorithm.

**Table 1:** Description and formula of hybrid deep neural network input.

Feature	Description	Formula
*f*_*ij*,1_^*∗*^	Position order [3]	*k*/*n*
*f*_*ij*,2_^*∗*^	Ratio of machine index *i* to machine number *m* [23]	*i*/*m*
*f*_*ij*,3_^*∗*^	Ratio of job index *j* to job number *n* [23]	*j*/*n*
*f*_*ij*,4_^*∗*^	Remaining processing time of job *j* [3]	(*T*_*j*_^cjp^ − *e*_*jk*_)/*T*_*j*_^cjp^
*f*_*ij*,5_^*∗*^	Ratio of operation processing time *p*_*ij*_ to total processing time [23]	*p*_*ij*_/*T*^total^
*f*_*ij*,6_^*∗*^	Ratio of operation processing time *p*_*ij*_ to processing time of machine *i* [23]	*p*_*ij*_/*T*_*i*_^cmp^
*f*_*ij*,7_^*∗*^	Ratio of operation processing time *p*_*ij*_ to processing time of job *j* [11]	*p*_*ij*_/*T*_*j*_^cjp^
*f*_*ij*,8_^*∗*^	Ratio of processing time of machine *i* to total processing time [11]	*T*_*i*_^cmp^/*T*^total^
*f*_*ij*,9_^*∗*^	Ratio of job *j*'s processing time to total job processing time	*T*_*j*_^cjp^/*T*^total^
*f*_*ij*,10_^*∗*^	Ratio of job *j*'s processing time to processing time of machine *i*	*T*_*j*_^cjp^/*T*_*i*_^cmp^

References indicate that this feature has been used in the corresponding literature.

**Table 2:** Processing time of the example 6 *∗* 8 JSSP.

	Position 1	Position 2	Position 3	Position 4	Position 5	Position 6
Job 1	15	26	18	11	25	12
Job 2	24	12	23	12	28	13
Job 3	22	29	12	27	20	15
Job 4	27	26	13	21	15	29
Job 5	11	29	21	12	24	18
Job 6	26	17	19	16	27	28
Job 7	24	29	18	27	14	23
Job 8	14	12	18	24	17	22

**Table 3:** Processing order of the example 6 *∗* 8 JSSP.

	Position 1	Position 2	Position 3	Position 4	Position 5	Position 6
Job 1	6	5	4	2	1	3
Job 2	3	6	2	1	4	5
Job 3	2	1	4	3	6	5
Job 4	2	1	3	5	4	6
Job 5	5	4	2	3	6	1
Job 6	3	6	2	4	5	1
Job 7	5	1	3	2	4	6
Job 8	4	6	3	1	2	5

**Table 4:** Data example of training deep scheduling neural network scheduler.

*i*	*j*	*k*	*Input*1	*Input*2						*Target*							
6	1	1	*F*_11_^*l*^	*D*_*P*^*l*^,*P*^*l*^_^2*d*^	*D*_*P*^*l*^,*E*^*l*^_^2*d*^	*D*_*P*^*l*^,*F*_11_^*l*^_^2*d*^	*D*_*E*^*l*^,*E*^*l*^_^2*d*^	*D*_*E*^*l*^,*F*_11_^*l*^_^2*d*^	*D*_*F*_11_^*l*^*F*_11_^*l*^_^2*d*^	1	0	0	0	0	0	0	0
5	1	2	*F*_12_^*l*^	*D*_*P*^*l*^,*P*^*l*^_^2*d*^	*D*_*P*^*l*^,*E*^*l*^_^2*d*^	*D*_*P*^*l*^,*F*_12_^*l*^_^2*d*^	*D*_*E*^*l*^,*E*^*l*^_^2*d*^	*D*_*E*^*l*^,*F*_12_^*l*^_^2*d*^	*D*_*F*_12_^*l*^*F*_12_^*l*^_^2*d*^	0	1	0	0	0	0	0	0
4	1	3	*F*_13_^*l*^	*D*_*P*^*l*^,*P*^*l*^_^2*d*^	*D*_*P*^*l*^,*E*^*l*^_^2*d*^	*D*_*P*^*l*^,*F*_13_^*l*^_^2*d*^	*D*_*E*^*l*^,*E*^*l*^_^2*d*^	*D*_*E*^*l*^,*F*_13_^*l*^_^2*d*^	*D*_*F*_13_^*l*^*F*_13_^*l*^_^2*d*^	0	0	1	0	0	0	0	0
3	1	4	*F*_14_^*l*^	*D*_*P*^*l*^,*P*^*l*^_^2*d*^	*D*_*P*^*l*^,*E*^*l*^_^2*d*^	*D*_*P*^*l*^,*F*_14_^*l*^_^2*d*^	*D*_*E*^*l*^,*E*^*l*^_^2*d*^	*D*_*E*^*l*^,*F*_14_^*l*^_^2*d*^	*D*_*F*_14_^*l*^*F*_14_^*l*^_^2*d*^	0	0	0	1	0	0	0	0
…	…	…	…	…	…	…	…	…	…	…	…	…	…	…	…	…	…
5	8	6	*F*_86_^*l*^	*D*_*P*^*l*^,*P*^*l*^_^2*d*^	*D*_*P*^*l*^,*E*^*l*^_^2*d*^	*D*_*P*^*l*^,*F*_86_^*l*^_^2*d*^	*D*_*E*^*l*^,*E*^*l*^_^2*d*^	*D*_*E*^*l*^,*F*_86_^*l*^_^2*d*^	*D*_*F*_86_^*l*^*F*_86_^*l*^_^2*d*^	0	0	0	0	0	0	0	1

**Table 5:** Output example of training deep scheduling neural network scheduler.

Job info			Output							
*i*	*j*	*k*	*p*(*x*_*ij*1_=1)	*p*(*x*_*ij*2_=1)	*p*(*x*_*ij*3_=1)	*p*(*x*_*ij*4_=1)	*p*(*x*_*ij*5_=1)	*p*(*x*_*ij*6_=1)	*p*(*x*_*ij*7_=1)	*p*(*x*_*ij*8_=1)
6	1	1	0.860	0.140	0.000	0.000	0.000	0.000	0.000	0.000
5	1	2	0.162	0.573	0.236	0.029	0.000	0.000	0.000	0.000
4	1	3	0.004	0.143	0.142	0.544	0.305	0.005	0.000	0.000
3	1	4	0.028	0.200	0.724	0.020	0.028	0.000	0.000	0.000
…	…	…	…	…	…	…	…	…	…	…
5	8	6	0.000	0.000	0.000	0.000	0.020	0.000	0.265	0.735

**Table 6:** Confusion matrix table of the ANN.

	Priority 1	Priority 2	Priority 3	Priority 4	Priority 5	Priority 6	Priority 7
Priority 1	621	66	11	0	0	0	2
Priority 2	35	543	85	12	5	1	19
Priority 3	30	70	436	96	17	21	30
Priority 4	5	14	153	357	74	24	73
Priority 5	2	6	11	198	363	62	58
Priority 6	7	1	4	34	176	457	21
Priority 7	0	0	0	3	65	135	497
Classification accuracy	0.89	0.78	0.62	0.51	0.52	0.65	0.71
Total accuracy	0.67						

**Table 7:** Confusion matrix table of the HDNNS.

	Priority 1	Priority 2	Priority 3	Priority 4	Priority 5	Priority 6	Priority 7
Priority 1	683	14	1	0	0	0	2
Priority 2	15	639	26	3	5	1	11
Priority 3	2	32	641	17	0	3	5
Priority 4	0	8	24	615	21	0	32
Priority 5	0	7	4	48	621	12	8
Priority 6	0	0	4	14	26	634	22
Priority 7	0	0	0	3	27	50	620
Classification accuracy	0.98	0.91	0.92	0.88	0.89	0.91	0.89
Total accuracy	0.91						

**Table 8:** Comparison of HDNNS with other methods on ZLP datasets.

		Optimal	HDNNS (our)	DQN	DEEPRM	HNN	ANN (1D)	ANN (all)	STPT	SPT
ZLP (8 *∗* 8) *p*∼*U* (10, 20)	Average MAKESPAN	156.82	174.25	177.80	178.51	190.52	195.52	182.52	181.58	201.11
	Scheduling score	—	90.0	88.2	87.9	82.3	80.2	85.9	86.4	78.0
	Training time	—	5396.2	12987.3	12568.6	—	427.6	9482.5	—	—
	Scheduling time	—	292.7	298.7	294.3	352.6	407.5	367.4	230.1	200.1

ZLP (8 *∗* 8) *p*∼*U* (10, 40)	Average MAKESPAN	269.93	305.69	306.39	309.05	331.85	338.21	313.54	324.19	347.45
	Scheduling score	—	88.3	88.1	87.3	81.3	79.8	86.1	83.3	77.7
	Training time	—	5124.5	13974.2	13213.8	—	425.8	9426.6	—	—
	Scheduling time	—	291.5	295.3	293.5	353.6	408.4	392.1	210.2	210.2

ZLP (8 *∗* 8) *p*∼*U* (10, 60)	Average MAKESPAN	386.43	428.41	436.15	434.14	458.84	486.07	449.62	464.49	488.51
	Scheduling score	—	90.2	88.6	89.0	84.2	79.5	85.9	83.2	79.1
	Training time	—	5135.8	13488.82	12846.5	—	424.5	9814.2	—	—
	Scheduling time	—	264.6	280.3	293.5	371.6	401.1	391.4	250.3	180.5

ZLP (8 *∗* 8) *p*∼*U* (10, 80)	Average MAKESPAN	502.89	562.52	570.81	561.26	623.69	591.64	590.15	593.12	632.95
	Scheduling score	—	89.4	88.1	89.6	80.6	85.0	85.2	84.8	79.5
	Training time	—	5217.7	13425.7	12681.6	—	425.4	9823.6	—	—
	Scheduling time	—	271.2	273.4	286.5	401.4	406.7	408.5	220.6	200.1

ZLP (13 *∗* 13) *p*∼*U* (10, 20)	Average MAKESPAN	290.58	332.85	332.47	331.63	357.67	345.92	338.69	380.97	402.98
	Scheduling score	—	87.3	87.4	87.6	81.2	84.0	85.8	76.3	72.1
	Training time	—	19162.9	61588.4	58584.7	—	424.0	37568.1	—	—
	Scheduling time	—	2384.5	2634.9	2871.5	5371.6	407.7	2589.4	2151.1	1840.1

ZLP (13 *∗* 13) *p*∼*U* (10, 40)	Average MAKESPAN	500.77	551.50	582.96	570.02	634.55	589.14	575.16	669.81	700.25
	Scheduling score	—	90.8	85.9	87.9	78.9	85.0	87.1	74.8	71.5
	Training time	—	20540.5	60974.3	58071.0	—	420.5	37481.6	—	—
	Scheduling time	—	2372.5	2628.4	2899.8	5375.4	407.0	2648.6	2284.4	1863.8

ZLP (13 *∗* 13) *p*∼*U* (10, 60)	Average MAKESPAN	720.57	794.45	819.76	814.20	905.10	885.22	869.26	868.37	913.87
	Scheduling score	—	90.7	87.9	88.5	79.6	81.4	82.9	83.0	78.8
	Training time	—	19346.4	64789.2	60325.7	—	425.1	39426.4	—	—
	Scheduling time	—	2384.8	2677.2	2987.7	5471.3	406.7	2468.7	2145.6	2056.1

ZLP (13 *∗* 13) *p*∼*U* (10, 80)	Average MAKESPAN	1026.57	1123.16	1210.57	1203.90	1311.87	1262.69	1255.47	1219.00	1299.63
	Scheduling score	—	91.4	84.8	85.3	78.3	81.3	81.8	84.2	79.0
	Training time	—	19741.3	60148.5	58247.7	—	425.8	40259.6	—	—
	Scheduling time	—	2346.9	2615.5	2884.1	5577.7	407.7	2945.4	2181.5	2054.1

AVE	Average MAKESPAN	481.82	534.10	554.61	550.34	601.76	586.80	571.80	587.69	623.34
	Scheduling score	—	89.8	87.3	87.9	80.8	82.0	85.1	82.0	77.0
	Training time	—	12458.2	37672.1	35817.5	—	424.8	24160.3	—	—
	Scheduling time	—	1556.1	1579.9	1601.4	2909.4	406.6	1526.4	1209.2	1075.6

Average MAKESPAN rank			1	3	2	7	5	4	6	8
Training time rank			2	4	3	—	1	5	—	—
Scheduling time RANK			5	6	7	8	1	4	3	2

**Table 9:** Comparison of HDNNS with other methods on traditional datasets.

		Optimal	HDNNS (our)	DQN	DEEPRM	ANN (1D)	ANN (all)	STPT	SPT
ft10 (10 *∗* 10)	MAKESPAN	930	1023	1023	1025	1154	1054	1152	1169
	Scheduling score	—	90.91	90.9	90.7	80.5	88.2	80.7	79.5
	Training time	—	1804.5	13321.7	12212.2	426.1	3245.6	—	—
	Scheduling time	—	3.6	3.9	3.8	1.2	4.7	1.0	1.0

ft20 (20 *∗* 10)	MAKESPAN	1165	1391	1342	1317	1524	1504	1434	1544
	Scheduling score	—	83.7	86.8	88.4	76.4	77.4	81.2	75.4
	Training time	—	3954.1	16532.1	17548.3	436.5	4689.5	—	—
	Scheduling time	—	7.6	7.4	7.2	2.4	9.2	1.9	1.9

la24 (20 *∗* 10)	MAKESPAN	935	1056	1088	1071	1564	1564	1580	1569
	Scheduling score	—	88.5	85.9	87.30	59.7	59.7	59.1	59.5
	Training time	—	3976.5	16844.3	16254.5	487.6	4684.4	—	—
	Scheduling time	—	7.6	8.2	7.3	2.5	2.5	1.9	1.9

la36 (15 *∗* 15)	MAKESPAN	1268	1318	1465	1465	1721	1721	1729	1729
	Scheduling score	—	96.2	86.55	86.5	73.6	73.6	73.3	73.3
	Training time	—	15318.1	63172.0	62251.4	578.5	21688.1	—	—
	Scheduling time	—	38.4	39.3	39.2	3.3	42.5	8.3	7.2

abz7 (20 *∗* 15)	MAKESPAN	665	726	739	720	940	940	980	1026
	Scheduling score	—	91.6	89.9	92.3	70.7	70.7	67.8	64.8
	Training time	—	22124.2	90584.4	92584.4	683.4	29258.3	—	—
	Scheduling time	—	51.3	48.3	50.24	4.8	89.5	13.3	12.3

yn1 (20 *∗* 20)	MAKESPAN	886	995	1183	1067	1183	1183	1208	1207
	Scheduling score	—	89.0	74.8	83.04	74.8	74.8	73.3	73.4
	Training time	—	30688.8	126689.2	125845.4	536.0	35648.2	—	—
	Scheduling time	—	177.2	188.0	184.5	65.4	194.5	78.4	73.5

Average	MAKESPAN	974.83	1084.83	1140.0	1110.8	1347.6	1327.6	1347.1	1374.0
	Scheduling score	—	90.01	85.5	88.0	72.6	74.1	72.6	71.0
	Training time	—	12977.7	54523.9	54449.4	524.7	16535.7	—	—
	Scheduling time	—	47.6	49.1	48.7	13.3	57.1	17.5	16.3

MAKESPAN rank			1	3	2	5	4	6	7
Training time			2	4	3	1	5	—	—
Scheduling time RANK			4	6	5	1	7	3	2

**Table 10:** Comparison tables with traditional classification methods on ZLP datasets.

		Near optimal	HDNNS (our)	ANN (1D)	KNN	SVM	DT	ERT	GOSS
ZLP (15 *∗* 12) *p*∼*U* (10, 40)	Average MAKESPAN	320.14	334.88	369.68	424.03	426.29	403.20	398.69	400.18
	Scheduling score	—	95.6	86.6	75.5	75.1	79.4	80.3	80

ZLP (15 *∗* 12) *p*∼*U* (10, 50)	Average MAKESPAN	375.04	398.55	434.57	498.72	512.34	482.67	464.73	472.34
	Scheduling score	—	94.1	86.3	75.2	73.2	77.7	80.7	79.4

ZLP (15 *∗* 12) *p*∼*U* (10, 60)	Average MAKESPAN	443.97	450.27	513.85	584.94	599.96	581.11	547.44	550.15
	Scheduling score	—	98.6	86.4	75.9	74	76.4	81.1	80.7

ZLP (15 *∗* 12) *p*∼*U* (10, 70)	Average MAKESPAN	513.13	551.16	591.16	676.95	686.00	645.45	636.64	635.85
	Scheduling score	—	93.1	86.8	75.8	74.8	79.5	80.6	80.7

ZLP (15 *∗* 12) *p*∼*U* (10, 80)	Average MAKESPAN	560.94	618.46	653.78	751.93	744.94	705.59	702.93	704.70
	Scheduling score	—	90.7	85.8	74.6	75.3	79.5	79.8	79.6

ZLP (15 *∗* 18) *p*∼*U* (10, 40)	Average MAKESPAN	476.40	508.43	550.75	617.90	649.05	606.88	597.74	599.25
	Scheduling score	—	93.7	86.5	77.1	73.4	78.5	79.7	79.5

ZLP (15 *∗* 18) *p*∼*U* (10, 50)	Average MAKESPAN	571.62	617.97	660.83	737.57	774.55	725.41	711.86	732.85
	Scheduling score	—	92.5	86.5	77.5	73.8	78.8	80.3	78

ZLP (15 *∗* 18) *p*∼*U* (10, 60)	Average MAKESPAN	681.60	745.73	789.80	898.02	926.09	874.97	846.71	858.44
	Scheduling score	—	91.4	86.3	75.9	73.6	77.9	80.5	79.4

ZLP (15 *∗* 18) *p*∼*U* (10, 70)	Average MAKESPAN	794.26	849.48	920.35	1034.19	1074.78	1024.85	990.35	1019.59
	Scheduling score	—	93.5	86.3	76.8	73.9	77.5	80.2	77.9

ZLP (15 *∗* 18) *p*∼*U* (10, 80)	Average MAKESPAN	900.40	971.31	1043.34	1176.99	1210.22	1151.41	1138.31	1151.41
	Scheduling score	—	92.7	86.3	76.5	74.4	78.2	79.1	78.2

Average	Average MAKESPAN	563.75	604.62	652.81	740.13	760.42	720.15	703.54	712.47
	Scheduling score	—	93.59	86.38	76.08	74.15	78.34	80.23	79.34

Average MAKESPAN rank			1	2	6	7	5	3	4

## Data Availability

The data used to support the findings of this study are available from the corresponding author upon request.
